# A Case of Toxic Shock Syndrome Following an Injury Sustained During Scuba Diving

**DOI:** 10.7759/cureus.86758

**Published:** 2025-06-25

**Authors:** Yoshitaka Saegusa

**Affiliations:** 1 Department of Surgery, Okinawa Prefectural Yaeyama Hospital, Ishigaki, JPN; 2 Department of Gastroenterological and Transplant Surgery, Graduate School of Biomedical and Health Sciences, Hiroshima University, Hiroshima, JPN

**Keywords:** hd (hemodialysis), intensive care unit, s. aureus, scuba diving, toxic shock syndrome (tss)

## Abstract

*Staphylococcus aureus* (*S. aureus*) is a common pathogen that resides as a commensal in humans. This pathogen can cause toxic shock syndrome (TSS), a severe condition characterized by sudden onset and rapid progression to multiple organ failure. The skin and soft tissues are common sites of primary infections. We report a rare case of a 51-year-old male, in whom TSS developed due to trauma to the knee sustained during scuba diving and successfully managed with intensive care, including appropriate antibiotic therapy and hemodialysis.

## Introduction

*Staphylococcus aureus* (*S. aureus*) is a common commensal organism found in the anterior nares, axillae, vagina, and perineum. A total of 30-50% of healthy adults are colonized [1]. In cases of injury or trauma, this colonization can allow the organism to invade deeper tissues and enter the bloodstream, potentially leading to severe systemic infections [2]. Approximately 25% of *S. aureus* strains produce toxins, such as enterotoxins and toxic shock syndrome toxin-1 (TSST-1), which can induce hypotension, high-grade fever, and diarrhea, often leading to life-threatening systemic conditions [3]. It is estimated that 4-10% of healthy individuals are persistent carriers of these toxin-producing strains [4]. This severe condition, caused by toxins, such as TSST-1, is referred to as toxic shock syndrome (TSS). While TSS can arise from *S. aureus* infections associated with injury, it is also notably linked to the use of medical devices like tampons, leading to menstrual TSS (mTSS) [5].

TSS is a rare and life-threatening disease characterized by sudden onset and rapid progression to multiple organ failure, even in otherwise healthy individuals [6]. This report details a case of TSS complicated by rhabdomyolysis and acute kidney injury, necessitating hemodialysis following a scuba diving-related injury.

## Case presentation

Herein, we present a case of a 51-year-old male with no significant medical history. He worked as an instructor in scuba diving and sustained an injury to his left knee after striking coral while diving. The following day, the patient developed swelling in the left knee and a fever. On the third day, he experienced fecal incontinence and difficulty walking, prompting a visit to our emergency department.

Upon arrival at the hospital, the patient showed mild impairment of consciousness. His vital signs were as follows: blood pressure 81/41 mmHg, pulse rate 126 beats per minute, body temperature 38.0°C, respiratory rate 28 breaths per minute, SpO_2_ 96% on room air, and a Glasgow Coma Scale score of E3V4M3.

**Figure 1:**
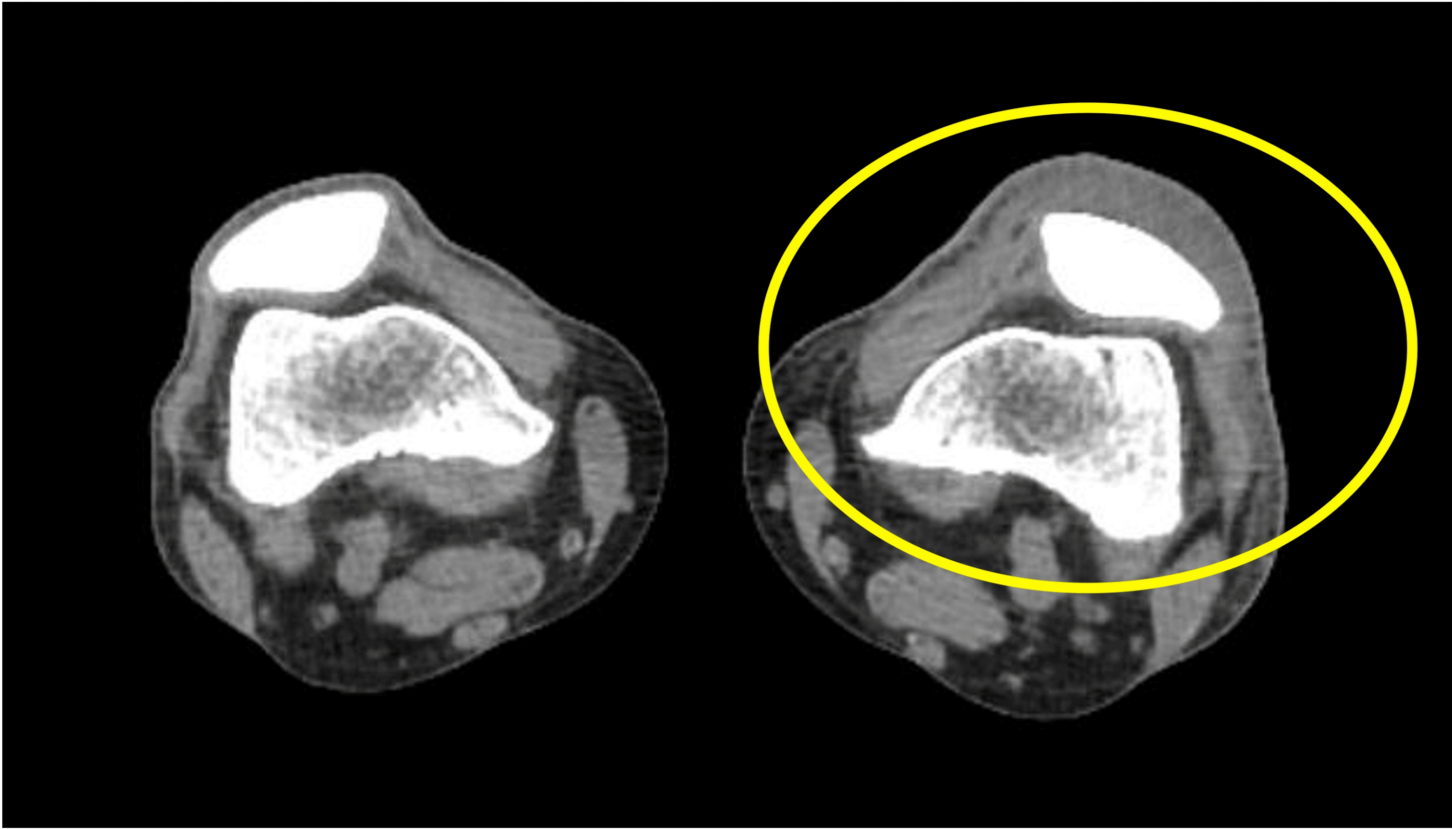
CT scan of the knee, demonstrating increased attenuation of the peri-knee adipose tissue in the left knee (circled in yellow) without any fluid collection or gas formation.

Physical examination revealed a wound on the left knee with clear signs of surrounding inflammation. Laboratory results on admission, summarized in Table 1, showed an increased leukocyte count, elevated C-reactive protein (CRP) and creatine phosphokinase (CPK) levels. It also showed coagulopathy and dysfunction of the liver and kidneys. Computed tomography (CT) showed increased attenuation of the peri-knee adipose tissue, with no detectable fluid collection or gas formation (Figure 1). The patient was diagnosed with cellulitis and septic shock and was admitted to the intensive care unit (ICU).

**Table 1:** Laboratory findings on admission. PT-INR: prothrombin time-international normalized ratio; APTT: activated partial thromboplastin time; Fib: fibrinogen; FDP: fibrin degradation products

Test	Values	Reference range
White blood cell (WBC) (per μL)	19,720	3,300-8,600
Hemoglobin (Hb) (g/dL)	14.6	13.7-16.8
Platelets (per μL)	157,000	158,000-348,000
Sodium (Na) (mmol/L)	133	138-145
Potassium (K) (mmol/L)	3.7	3.6-4.8
Chlorine (Cl) (mmol/L)	96	101-108
Blood urea nitrogen (BUN) (mg/dL)	36.0	8-20
Creatinine (mg/dL)	2.99	0.65-1.07
Total protein (g/dL)	6.5	6.8-8.3
Albumin (g/dL)	3.3	3.8-5.4
Total bilirubin (mg/dL)	2.7	0.4-1.5
Aspartate aminotransferase (AST) (U/L)	168	13-30
Alanine transaminase (ALT) (U/L)	181	10-42
Alkaline phosphatase (ALP) (U/L)	91	38-113
Lactate dehydrogenase (LDH) (U/L)	512	124-222
γ-Glutamyl transpeptidase (γ-GTP) (U/L)	282	13-64
Creatine kinase (CPK) (U/L)	2,212	59-248
C-reactive protein (CRP) (mg/dL)	26.71	0.00-0.14
PT-INR	1.63	0.9-1.1
APTT (s)	41.6	27.1-40.9
Fib (mg/dL)	648	150-400
FDP (µg/dL)	22	<10
D-dimer (µg/mL)	8.6	<1.0

**Figure 2:**
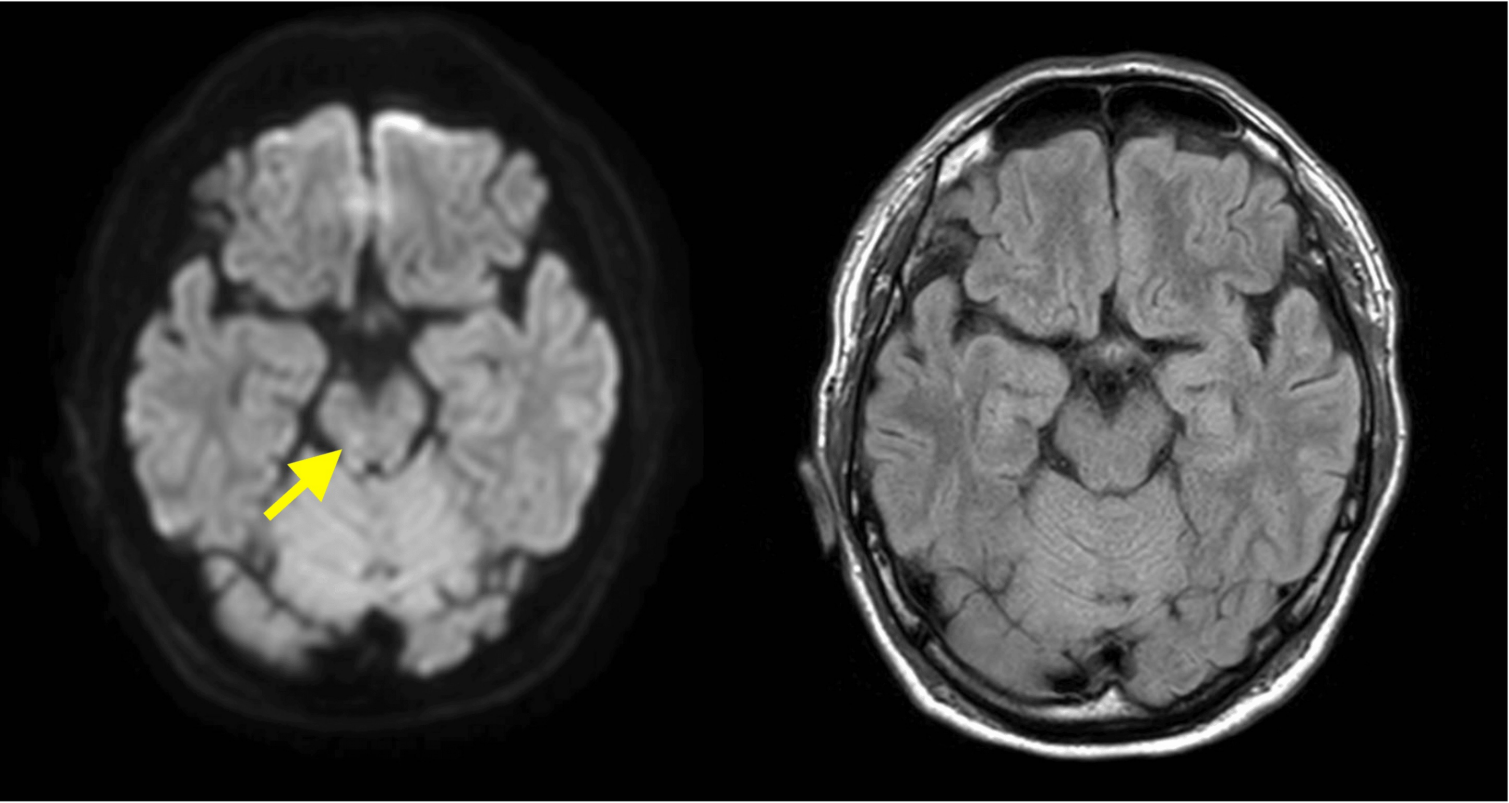
Diffusion-weighted imaging (DWI) on brain MRI (left) shows a midbrain infarction indicated by a yellow arrow. Fluid-attenuated inversion recovery (FLAIR) imaging (right) does not clearly demonstrate the infarction.

Meropenem and vancomycin were initiated as initial therapies; however, the patient developed diarrhea and a skin rash on the trunk after admission. The body temperature elevated to 40.9°C. Based on the clinical course, TSS was suspected, and clindamycin was added to the treatment regimen. On the second day of hospitalization, the patient exhibited dysarthria. Brain magnetic resonance imaging (MRI) revealed a midbrain infarction (Figure 2). Septic emboli were suspected, and three sets of blood cultures were obtained, all of which yielded negative results.

**Figure 3:**
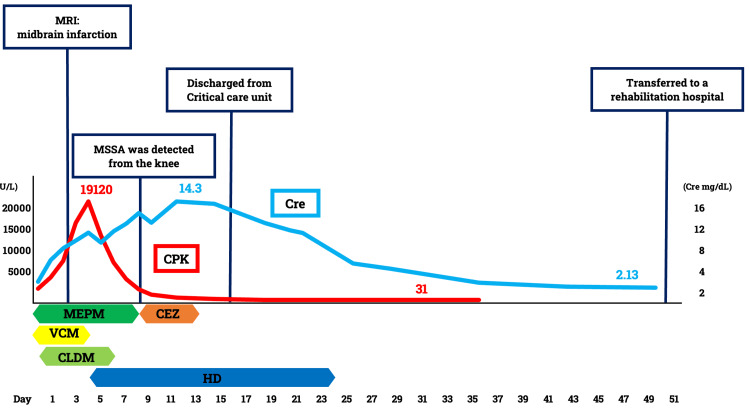
Clinical course of the patient. The graph illustrates trends in creatine kinase (CPK) and creatinine (Cre) levels over time, along with key medical interventions and events. Initial CPK levels peaked at 19,120 U/L, and the maximum creatinine level reached 14.3 mg/dL. Hemodialysis (HD) was initiated on day four and discontinued on day 24. The patient was transferred to a rehabilitation hospital on day 50. The graph also shows the duration of administration of the antibiotics meropenem (MEPM), vancomycin (VCM), clindamycin (CLDM), and cefazolin (CEZ), as well as the duration of HD. MSSA: methicillin-susceptible *Staphylococcus aureus*

CPK levels continued to rise, reaching 19,120 U/L, accompanied by oliguria and worsening pulmonary congestion. Hemodialysis was initiated on day four of hospitalization. The patient’s condition gradually improved, and dialysis was discontinued on hospital day 24. Although mild dysarthria persisted due to the cerebral infarction, the patient regained the ability to walk and was transferred to a rehabilitation facility on hospital day 50 (Figure 3).

*S. aureus* was isolated from a wound on the left knee. Antimicrobial susceptibility testing revealed that the isolate was methicillin-susceptible *Staphylococcus aureus* (MSSA) (Table 2). Although a specific toxin detection test, such as for TSST-1, was not performed due to institutional limitations, the diagnosis of TSS caused by *S. aureus* was made based on the characteristic clinical course. We compared the patient’s presentation with the CDC diagnostic criteria for TSS [7]. Of the 13 criteria, nine were met, including fever, diffuse macular erythroderma, desquamation, hypotension, involvement of the gastrointestinal tract, liver, muscle, and kidneys, along with negative blood cultures. One criterion could not be evaluated due to a lack of testing (Table 3).

**Table 2:** Antimicrobial susceptibility test.

Antimicrobial agents	MIC (μg/mL)
Oxacillin	0.25
Cefalexin	2
Penicillin G potassium	>2
Sulbactam/ampicillin	2
Cefazolin	8
Cefmetazole	2
Vancomycin	2
Linezolid	2
Daptomycin	0.5
Ciprofloxacin	≤0.25
Minocycline	≤0.25
Cefotaxime	4
Clindamycin	≤0.12
Erythromycin/clindamycin	≤4
Imipenem/cilastatin	≤0.25
Trimethoprim/sulfamethoxazole	≤1.25
Doxycycline	≤2
Rifampicin	≤0.5

**Table 3:** The criteria for toxic shock syndrome (other than streptococcal) on CDC 2011. The criteria for toxic shock syndrome (other than streptococcal) as of 2011, according to the CDC [8], are as follows: probable - a case meeting the laboratory criteria in which four of the five clinical criteria described above are present. Confirmed - a case that meets the laboratory criteria and in which all five of the clinical criteria described above are present, including desquamation, unless the patient dies before desquamation occurs. TSS: toxic shock syndrome; T-Bil: total bilirubin; AST: aspartate aminotransferase; ALT: alanine transaminase

Criteria of TSS on CDC2011	Present case	Criteria met
Clinical criteria		
Fever: greater than or equal to 38.9°C	40.9°C	Yes
Rash: diffuse macular erythroderma	Rash on the trunk	Yes
Desquamation: 1-2 weeks after onset of rash	Desquamation on the feet during treatment	Yes
Hypotension: systolic blood pressure less than or equal to 90 mmHg for adults	81 mmHg	Yes
Multisystem involvement (three or more of the following organ systems)	-	Yes
Gastrointestinal: vomiting or diarrhea at onset of illness	Diarrhea	Yes
Mucous membrane: vaginal, oropharyngeal, or conjunctival hyperemia	None	No
Muscular: severe myalgia or creatine phosphokinase level at least twice the upper limit of normal	CPK: 19,120 U/dL	Yes
Renal: blood urea nitrogen or creatinine at least twice the upper limit of normal for laboratory or urinary sediment with pyuria (greater than or equal to 5 leukocytes per high-power field) in the absence of urinary tract infection	Cre: 14.3 mg/dL	Yes
Hepatic: T-Bil, AST, or ALT levels at least twice the upper limit of normal for laboratory	T-Bil 2.7 mg/dL, AST 181 U/L, ALT 91 U/L	Yes
Hematologic: platelets less than 100,000/mm^3^	157,000/mm^3^	No
Central nervous system: disorientation or alterations in consciousness without focal neurologic signs when fever and hypotension are absent	Not applicable (due to suffering cerebral infarction)	No
Laboratory criteria		
Negative for blood or cerebrospinal fluid cultures	Negative for blood culture	Yes
Negative serologies for Rocky Mountain spotted fever, leptospirosis, or measles	Not examined	N/A

## Discussion

This case highlights the rare occurrence of TSS precipitated by a scuba diving-related coral injury, leading to severe rhabdomyolysis and acute kidney injury requiring hemodialysis. While *S. aureus* is a well-established cause of TSS, the specific context of a coral-induced wound in a healthy diving instructor warrants particular attention.

The patient's clinical presentation was characterized by rapid progression to shock, rash, diarrhea, and multi-organ failure, including acute kidney injury and liver dysfunction, consistent with TSS criteria [7]. The initial diagnosis of cellulitis and septic shock, which led to admission to the ICU, was expanded to suspect TSS following the development of diarrhea, skin rash, and high-grade fever post-admission. Despite negative results from three sets of blood cultures, *S. aureus* was isolated from the knee wound, confirming the diagnosis of TSS. This absence of bacteremia, while seemingly counterintuitive, is not uncommon in TSS, as the systemic effects are primarily mediated by bacterial exotoxins rather than by direct bacterial dissemination [4]. In this case, the patient developed midbrain infarction during the clinical course, presumed to result from a hypercoagulable state induced by a cytokine storm and hypotension.

Although specific testing for TSST-1 or other superantigenic toxins was not performed due to institutional limitations, the diagnosis of TSS was supported by the patient’s clinical course and fulfillment of the CDC diagnostic criteria. We acknowledge this as a limitation of our case.

TSS was first reported in 1978 as a pediatric disease [9]. Although the number of reported cases has increased, it remains rare, with an estimated incidence of 1-2 per 100,000 population. The causative toxin, TSST-1, is a superantigen that cross-links major histocompatibility complex (MHC) molecules and T-cell receptors (TCRs) in an antigen-independent manner. This process leads to non-specific T-cell activation and a subsequent cytokine storm that can result in severe illness [10].

This case emphasizes the importance of considering TSS in patients presenting with systemic symptoms following any skin injury, particularly those sustained in aquatic environments during scuba diving. To the best of our knowledge, there have been no previous reports of TSS caused by trauma related to scuba diving. A reported case of *S. aureus* infection was attributed to coral dermatitis [11]. Clinicians should be aware that TSS can develop rapidly even in patients without typical risk factors and requires immediate recognition and intervention [12]. Early administration of antibiotics, especially those with antitoxin effects such as clindamycin, along with robust supportive care, is crucial for improving patient outcomes [13].

## Conclusions

Clinicians should consider TSS associated with *S. aureus* when a patient presents with an injury, including those resulting from diving. This case highlights that TSS can develop rapidly, even in patients without typical risk factors, and emphasizes the need for immediate recognition and intervention.

## References

[1] Lowy FD (1998). Staphylococcus aureus infections. N Engl J Med.

[2] Thomer L, Schneewind O, Missiakas D (2016). Pathogenesis of Staphylococcus aureus bloodstream infections. Annu Rev Pathol.

[3] Wilkins AL, Steer AC, Smeesters PR, Curtis N (2017). Toxic shock syndrome - the seven Rs of management and treatment. J Infect.

[4] Kawahara N, Irimura K, Uchimura C, Ogata M (2019). A case of toxic shock syndrome from an abrasion caused by a motorcycle accident. Jpn J Med Technol.

[5] Schlievert PM, Davis CC (2020). Device-associated menstrual toxic shock syndrome. Clin Microbiol Rev.

[6] Hansen NS, Leth S, Nielsen LT (2020). Toxic shock syndrome. [Article in Danish]. Ugeskr Laeger.

[7] Atchade E, De Tymowski C, Grall N, Tanaka S, Montravers P (2024). Toxic shock syndrome: a literature review. Antibiotics (Basel).

[8] Centers for Disease Control and Prevention (CDC). (2011 (2025). Toxic shock syndrome (other than Streptococcal) (TSS) 2011 case definition. https://ndc.services.cdc.gov/case-definitions/toxic-shock-syndrome-2011/.

[9] Todd J, Fishaut M, Kapral F, Welch T (1978). Toxic-shock syndrome associated with phage-group-I Staphylococci. Lancet.

[10] Fukuuchi F, Hida M, Fujimoto Y, Hiraga S, Satoh T (1996). A case of toxic shock syndrome (TSS) induced by methicillin-resistant staphylococcus aureus (MRSA) presenting as acute renal failure with disseminated intravascular coagulation. [Article in Japanese]. Nihon Jinzo Gakkai Shi.

[11] Kandi V (2018). Coral dermatitis or infectious dermatitis: report of a case of Staphylococcus aureus infection of skin after scuba diving. Cureus.

[12] Ishola F, Mangat GK, Martinez K, Mohammed YN, McKany M (2023). Atypical case presentation of toxic shock syndrome. Cureus.

[13] Lappin E, Ferguson AJ (2009). Gram-positive toxic shock syndromes. Lancet Infect Dis.

